# Carpal Tunnel Syndrome and Calcium Deposit in the Surgically Transacted Transverse Carpal Ligament

**DOI:** 10.1155/2022/2864485

**Published:** 2022-04-07

**Authors:** Ali Abbas Hashim Al-Musawi, Nadia Mahmoud Tawfiq Jebril, Amer Abbas. H. AL-Auhaimid, Zaid Hadi. K. Hammoodi

**Affiliations:** ^1^Consultant Neurosurgeon FRCS Glasg, FRCSED, FRCSE, FACS, FIBMS, University of Babylon, Babylon, Iraq; ^2^Department of Neurosurgery, Hammurabi College of Medicine, University of Babylon, Babylon, Iraq; ^3^Department of Biology, College of Sciences for Women, University of Babylon, Babylon, Iraq; ^4^Department of Orthopaedic Surgery, Imam Al-Sadiq Teaching Hospital, Babylon, Iraq; ^5^Department of Radiology, Baghdad Medical City, Shaheed Al Hariri Surgical Speciality Hospital, Baghdad, Iraq

## Abstract

A diagnosis of carpal tunnel syndrome (CTS) in a human often contains more than one test. Calcification of the traverse carpal ligament (TCL) is the common reason why patients seek CTS surgery. However, the determination of calcium (Ca) concentration in the TCL has not been studied. The results of environmental toxicity studies assessing the relationship between Ca and elemental deposition in the TCL are inconsistent. The purpose of this paper was to verify this hypothesis by conducting a chemical analysis of a portion of the released TCL to assess whether there is a relationship between CTS and Ca, measured as the total concentration of Ca, and to measure the precipitation of elements; the most closely related elements associated with Ca are cadmium and lead, which are also toxic. Surgical release of TCL was performed on forty patients. Total concentrations of Ca, Cd, and Pb in the extracted portion of TCL were digested and determined using inductively coupled plasma mass spectrometry (ICP-MS) and the possibility of using X-ray spectroscopy (XRF) for direct elemental analysis. Ca mineralisation was revealed in some TCLs. In assessing patients' environmental pollution, it was observed that the Cd and Pb concentrations were significant with a higher Ca concentration, and XRF was useful for direct detection of the elements in samples of the human body. These results indicate that TCL mineralisation by Ca does not characterise CTS, which has important concerns in improving patients' therapeutic strategies, and Cd and Pb concentrations varied due to different factors.

## 1. Introduction

CTS surgery in Iraq is the most common operation [1, 2]. CTS surgery involves expanding the size of the carpal tunnel and reducing the pressure on the median nerve by releasing the transverse carpal ligament (TCL). The reasons for patients requesting this type of surgery could be related to their job behaviour. CTS is the most common cause of pain in the human hand due to trauma, acute rheumatoid arthritis, spontaneous bleeding, burning, and purulent or viral infection. Usually, there is no standard method for diagnosing CTS [3]; a physical examination (electrophysiology test) is used to examine the affected nerve between the TCL by stimulating muscle action potentials to compound motor action potential (CMAP) to reflect the state of the motor fibres in the median nerve [4]. The incidence of CTS increases with frequent use of the hand, in addition to other factors such as gender, age, and weight [5]. Calcification in the TCL is known to cause acute CTS diagnosed by the standard method (X-rays), and calcium deposition in the wrist of people with CTS has been reported [6]. Mainly, calcium phosphate (C.P.), calcium pyrophosphate dihydrate (CPPD), calcium oxalate (C.O.), and monosodium urate (MSU) are the main calcified crystals in the tissue [7]. The clinical link of TCL crystals with calcium was not understood in CTS patients. Since the X-ray examination of TCL cannot diagnose whether or not calcified crystals are responsible for CTS, instead, it will be possible to measure the Ca concentration in the TCL as CPPD has been studied in the synovium and cartilage [8, 9]. There may be other reasons for getting symptoms of CTS, such as end-stage kidney disease (ESRD). It was found that patients with ESRD were more likely to undergo this type of surgery [10]. Causes of ESRD are related to other diseases such as diabetes (type 1 or type 2), high blood pressure, glomerulonephritis, interstitial nephritis, polycystic kidney disease, prolonged urinary obstruction (as a result of some types of cancer, prostate enlargement, and kidney stones), vesicoureteral reflux, or recurrent kidney infection [11]. In respect to ecotoxicological studies, human exposure to nephrotoxicants (such as Cd and Pb) causes ESRD [12]. Most epidemiological studies of human exposure to Cd have shown that the main target organs for Cd toxicity are the liver and kidneys. They contain the highest concentration of the protein metallothionein, which binds with Cd and concentrates it [13]. Cd toxicity is usually determined by measuring its concentration and biologically effective dose at the target organ. An Australian study showed Cd concentrations in the kidney cortex and liver were 26 and 1 (*μ*g Cd/g wet weight, respectively) [14]. Those epidemiological studies contribute to evaluating pollution in a region. Assessments of element concentrations in the human body are used as the common purpose of monitoring in ecotoxicological studies. Importantly, ecotoxicological studies are the most effective way to verify the association of measured elements in the human body with other diseases when patients live in contaminated areas. Therefore, the current study assumed that the measurement of Cd and Pb in the TCL fraction could be correlated with the deposing of Ca in the TCL. The cause of the association between calcification and Cd or Pb is due to the immobilisation of Cd and Pb with calcium phosphate (Ca^2+^PO_4_^3−^) as Cd_3_(PO_4_)_2_ (solubility constant, *K*_sp_ = 2.53 × 10^−33^) and PbHPO_4_ (*K*_sp_ = 10^−23.8^), respectively [15], which means the disproportion of Ca with Pb and Cd [16]. TCLs were obtained from forty patients who have been suffering from CTS. This study evaluated whether patients with CTS were associated with CPPD (diagnosed by measuring Ca in the TCL). The relationship between measured concentrations of Cd and Pb in the TCL and Ca released distinguish CTS patients from healthy controls. For comparative analysis, XRF analysis was performed. The prospective user of XRF for analysing elements within the human body was discussed using XRF for direct determination of the elements within the human organism in the medicine. To our knowledge, this is the new analysis of XRF performed on a human body to date [17, 18].

## 2. Methods

### 2.1. Carpal Tunnel Release (CTR) Surgery

**Figure 1:**
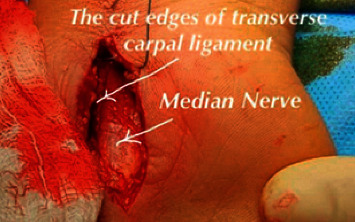
CTR surgery by the first author.

A day-case surgery on forty patients who suffered from CTS was operated on to relieve the symptoms of CTS. The operations were performed under local anaesthesia using acid-washed and sterilised surgical tools, as shown in Figure 1.  Table 1 summarises the demographic information and hand side affected by CTS of studied patients.

10% povidone/iodine solution (*w*/*v*) was applied to the skin of the palmar side of the wrist and the palm, and 10 mL of the anaesthetic agent lidocaine (solution 2% *w*/*v*) was applied to the palmar side of the wrist and the proximal part of the palm using local infiltration. CTR surgery involved cutting the median palmar skin using a surgical scalpel blade, followed by a self-retaining retractor insertion, and the subcutaneous tissue was dissected with the mosquito forceps and dissector. Then, the thick calcified transverse carpal ligament was excised with a surgical scalpel blade, and the median nerve was freed. Meanwhile, a small portion of the TCL was taken to achieve the objectives of this study. Control samples of TCL were taken from volunteers who had not suffered from and diagnosed with CTS. These control samples were used for assessing the Ca concentration in the TCL for comparison with the Ca concentration in the TCL of studied patients with CTS. Control samples of TCL were obtained from volunteers (*n* = 3). Briefly, volunteers were set in a prone position, and their wrists were placed on the examination couch with the palm facing forward. Scanning (with an iU22 scanner, *L* 7–13 MHz linear; Philips Medical Systems, USA) of the carpal tunnel of interest with localisation of critical anatomical structures, including the TCL, median nerve, ulnar artery, superficial digital flexor tendons, deep, scaphoid, hamate, and pisiform bones, was performed. Under sterilised conditions (10% povidone/iodine solution (*w*/*v*)), the anaesthetising of the skin was performed by injecting 2 mL of 2% lidocaine and 1 mL of isotonic saline with a fine 26-gauge needle. The sample of the TCL was taken under an ultrasound guide to avoid injury to the median nerve or flexor tendons. A Tru-Cut biopsy needle 20 gauge attached to a 5 mL syringe with coaxial needle (Disposable Core Biopsy Instrument; 20 G × 16 cm) was inserted to take a Tru-Cut biopsy from the medial hamate-pisiform side of TCL using the real-time ultrasound transducer in a longitudinal plane about 10–20° with the skin, and two small pieces were taken, each about 10–14 mm length and 0.5 mm width. Finally, the needle is pulled out with gentle pressure applied. After TCL extraction, ligaments were snap-frozen in liquid nitrogen and stored at −20°C for further study.

### 2.2. Determination of Ca, Cd, and Pb Concentrations in TCLs Using ICP-MS

Determination of Ca, Cd, and Pb concentrations in TCL was performed after *aqua regia* digestion, according to Lehner et al. [19]. The *aqua regia* solution was obtained by mixing one volume of nitric acid (>68%) with three volumes of hydrochloric acid (<37%), and the development of the golden colour was observed after a few minutes. The three wet sample ligaments (30 mg) from each patient was transferred to a Falcon tube (50 mL), and the digestion of the samples was done by adding 10 mL of the *aqua regia* and left for 24 h. After digestion, the volume to 50 mL was completed with 0.2% nitric acid (v/v). Indium was added into samples with a final concentration of 0.5 *μ*M as an internal standard before analyses. The concentrations of Ca, Cd, and Pb were analysed using ICP-MS (*X* Series 2; Thermo Scientific). ICP-MS was validated by evaluating some analytical parameters such as linearity, the limit of detection (LOD), limit of quantification (LOQ), precision (relative standard deviation, RSD), and accuracy. Linearity was assessed for the analytical response from the standard solution used during the measurement. The ranges of standard solution were from 10 to 400 mM for Ca and 10–400 *μ*M for both Cd and Pb. From plotting the intensity values (*y*-axis) of standard solutions versus their concentrations (*x*-axis), linear regression, interceptions, and % intercept were obtained (Table 2). The analytical response to the standard solutions as linear as *R*^2^ was above 0.995. The intercept percentage was low, which means no systematic error in the linear regression.

The analytical susceptibility of ICP-MS was estimated from the value of LOD and LOQ, which are the minutest concentration that ICP-MS can detect. The LODs of Ca, Cd, and Pb were established from the five times the standard deviation (S.D.) of measuring the lowest standard (*n* = 10) [12], at 0.02 mM, 0.01, and 0.01 *μ*M, respectively. While the LOQs of Ca, Cd, and Pb were calculated from the 3.3 times the S.D. of measuring the lowest standard (*n* = 10, according to [19]) and were 0.066 mM, 0.033 *μ*M, and 0.033 *μ*M, respectively. Based on LOD values, the instrument was sufficiently sensitive to the Ca, Cd, and Pb analysis because the LOD values were lower than the concentrations analysed in the previous studies in humans [20–22], respectively. Precision expressed as RSD of ICP-MS was evaluated by measuring the lowest standard (*n* = 3), and the RSD was calculated from 100 times the S.D. dividing by the average concentration. The results of RSD of Ca, Cd, and Pb (0.4, 0.5, and 0.1, respectively; obtained from the first day) showed good precision as the percentage of RSD value was less than five. For the determination of the intermediate precision, the same standards were measured again on the fourth and sixth day, and the RSDs were 1.3% (Ca), 1.0% (Cd), and 0.4% (Pb), as shown in Table 3.

Yttrium at a concentration of 0.5 *μ*M was used as an external standard during the measurement. As for the carpal ligament, there is no certificate reference material (CRM) market, which could be used to check digestion and analysis accuracy; therefore, a spike recovery test was performed [23]. The spike recovery test was carried out by spiking the ddH_2_O (1 mL) with nominal concentrations of Ca, Cd, and Pb (50 *μ*M, *n* = 3 samples), followed by *aqua regia* digestion. Meanwhile, *aqua regia* digestion was carried out with the same ddH_2_O (1 mL) before spiking Ca, Cd, and Pb. The spike recovery (percentage) was determined using the equation:

(Element measured in spiked ddH_2_O − Element measured in unspiked ddH_2_O)/(Element spiked) × 100.

The element measured in the spiked ddH_2_O is the total concentrations of elements determined at the end of the spike recovery test. In contrast, the element concentrations determined in the unspiked ddH_2_O are the total concentrations of elements measured before adding elements. The element spiked is the nominated concentrations of elements spiked into the ddH_2_O. Results of the Ca, Cd, and Pb spike test for the ddH_2_O (recoveries: 95 ± 0.02%, 99 ± 0.01%, and 93 ± 0.03%, respectively) are presented in Table 4, showing acceptable recoveries considering a standard range of 80–110% [23]. In the ICP-MS analysis, the procedural blanks (ddH_2_O or *aqua regia*) were used to correct the measured concentrations of elements in samples of ddH_2_O or TCL, respectively.

### 2.3. Determination of Ca, Cd, and Pb Concentrations in TCLs Using XRF Spectrometry

XRF spectrometry (NITON XL31EXRF) was used along with the calibration of biological certified reference material (bovine liver, NIST1577C; Sigma-Aldrich), and the duration of the reading was 120 s. Samples of the part of TCL were cut in thickness and length 1 cm; then the samples were placed in XRF cups (*n* = 3 samples), covered with a thin polypropylene film (4 µm) placed above the window of XRF. The measurement was performed in triplicate, and in each analysis, the position and orientation of the cup were changed. The accuracy percentage values were estimated from the recorded and certified standard reference material, bone ash (SRM 1400; Sigma-Aldrich). The recovery percentage of XRF for Pb was (98 ± 0.3%), indicating good accordance between the measured and certified value.

### 2.4. Statistical Analysis

All the data of the experiments were in the mean and standard error of the mean. Sigma Plot (version 13) and Excel illustrate the data. Statistical analyses, one-way or two-way ANOVA followed by Tukey's *post hoc* test, were performed on the data of concentrations in IBM SPSS Statistics 22 software to evaluate whether the concentrations of Ca, Cd, and Pb in the TCL were different between 40 patients. The concentrations of Ca, Cd, and Pb in the TCL were statistically analysed to find a significant difference between XRF and ICP-MS techniques. Correlation tests (Pearson) were performed on the measured concentrations of Ca, Cd, and Pb in the TCLs to determine if Cd or Pb concentrations were affected by the content of Ca in each patient.

## 3. Results

### 3.1. Concentrations of Ca, Cd, and Pb in TCL Determined by ICP-MS

**Figure 2:**
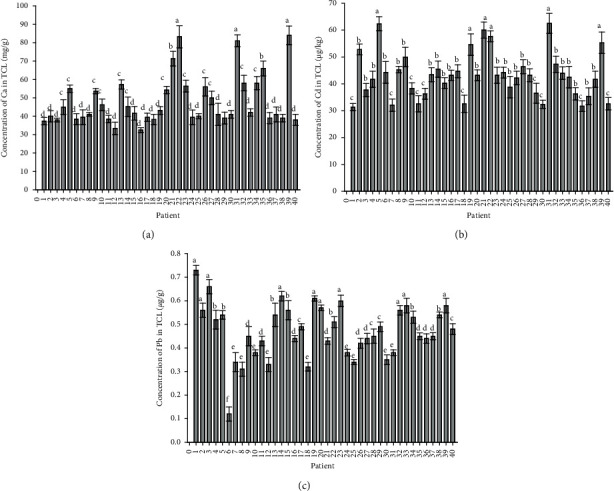
ICP-MS analyses were performed on samples (*n* = 3 and error bars are standard error of the mean) from the TCLs of 40 patients with CTS: (a) concentration of Ca, (b) concentration of Cd, and (c) concentration of Pb. The concentration of Ca in control samples was 37 ± 0.2 mg/g. The concentrations were subjected to two-way ANOVA and Tukey's *post hoc* test, and different letters on the bars indicate a significant difference in the concentrations between the patients.

The mean concentrations of Ca, Cd, and Pb analyses of carpal ligaments are summarised in Figure 2. The mean concentration of Ca in the controlled TCL extracted from healthy people was 37 ± 0.2 mg/g. The measured concentration of Ca in the TCLs of patients with CTS, as presented in Figure 2(a), shows that the Ca concentration has a broad distribution, and the statistical differences were observed. It is possible to note that the concentrations of the Ca are higher in some samples of TCLs than in control samples of TCLs. After measuring Ca in the TCL, the possible association between calcification and Cd or Pb deposition was determined. We focused on Cd and Pb as these elements could be precipitated with calcified crystals in the TCL. Cd concentrations in TCL patients are reported in Figure 2(b). Concentrations were markedly varied; the range of Cd content was between 31.4 ± 1.3 and 62.5 ± 3.7 *μ*g/g. In comparison, from Figure 2(c), it can be seen that Pb concentrations in the TCL of CTS patients were lower than Cd concentrations (ranging from 0.12 ± 0.03 to 0.73 ± 0.02 *μ*g/g).

### 3.2. Correlations between Ca and Cd or Pb Concentrations

**Figure 3:**
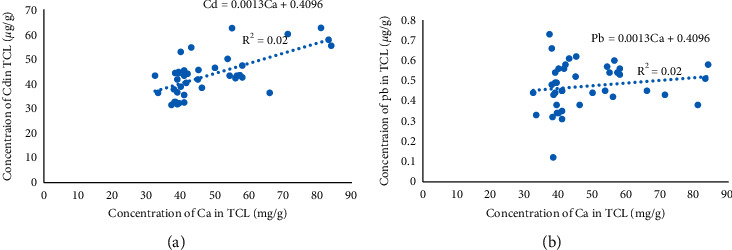
Correlations between measured concentrations of Ca and (a) Cd or (b) Pb in the TCLs of 40 patients with CTS.

The relationship between Ca and Cd is illustrated in Figure 3(a), which shows a moderate positive correlation between the concentration of Cd and Ca content in the TCL (correlation coefficient = 0.66, *p* < 0.001). The correlation between Pb and Ca suggested a minor decrease in Pb concentration as a result of increasing Ca concentrations in TCLs of CTS patients (correlation coefficient = 0.15, *p* < 0.001; Figure 3(b)).

### 3.3. Comparison between Two Techniques for Analysis of Ca, Cd, and Pb Concentrations in TCLs

**Figure 4:**
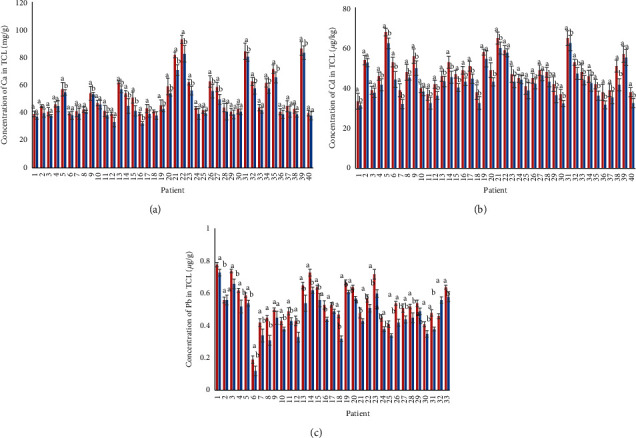
Comparison of (a) Ca, (b) Cd, and (c) Pb concentrations in the TCLs of 40 patients with CTS measured by XRF (red column) and ICP-MS (blue column) techniques. The concentrations were subjected to two-way ANOVA and Tukey's post hoc test, and different letters on the bars indicate a significant difference in the concentrations between the techniques. Error bars are the standard error of the mean.

XRF analysed directly the measurement of Ca, Cd, and Pb concentrations in TCLs.  Figure 4 shows the comparison between measuring Ca, Cd, and Pb concentrations in TCLs by XRF and ICP-MS, and differences in some samples show concentrations are more significant (^*a*^*p* < 0.05) in using the XRF technique. The main finding is the detection of Ca, Cd, and Pb concentrations in TCLs by XRF. Vanhoof et al. [24] stated that measurement with XRF technology gives a higher concentration of elements than ICP-MS. These results were due to samples from an analysis by ICP-MS being digested by acid to release the component, and sometimes digesting acid is insufficient to release elements [25].

## 4. Discussion

A total of 120 TCL samples were analysed, obtained from forty patients who suffered from CTS divided into three groups according to their age. There were 29 females and 11 males involved. Whenever the calcium investigation was performed in the TCL of patients with CTS, calcium determination was initially performed in the controlled TCL extracted from healthy people. This control was measured to compare the concentration of Ca in control and patient samples. The concentration of Ca in control was consistent with previous studies on calcium contents in human tissues [26]. The higher concentrations of Ca in some samples of TCLs than in control samples of TCLs suggest that the detection of Ca in the TCLs of CTS patients is not necessary because the differences in Ca contents may be caused by several factors, such as age and different disease stages [23]. Therefore, we did not indicate that Ca concentration affected the patients with CTS. The possible association between calcification and Cd or Pb deposition was varied. Several factors may explain this difference, such as exposure to pollution, age, gender, and smoking habit [14]. The results of Cd showed a good agreement with published data [18], and it was lower than the recorded concentration of Ca. This low concentration could be caused by having a *K*_sp_ of 10^−23.8^ of PbHPO_4_, lower than the *K*_sp_ of Cd_3_ (PO_4_)_2_ (2.53 × 10^−33^) [15]. In addition, the level of exposure to Pb pollution, metabolism process, and age also affect Pb content in the human body [27]. Pb concentration data in human tissues from this study are comparable to the study of Mari et al. [21], who reported that the concentration of Pb in the kidney was 0.18 *μ*g/g. Recently, García et al. [28] have estimated the concentration of Pb in human tissues (liver, kidney, brain, lung, and bone) with a range of 0.08 to 1.0 *μ*g/g. In the next step, we attempted to find the correlation between Ca and Cd or Pb concentrations in the TCL. Concerning studies conducted on the human body to measure element concentration, especially Ca, Cd, and Pb elements in Iraq, according to a systematic review and meta-analysis by Saghzadeh and Rezaie [29], Iraq does not have any such study. The moderate positive correlation between the concentration of Cd and Ca content in the TCL indicates that the contents of Ca in TCLs of CTS patients may positively affect Cd concentrations and enable it to precipitate. The correlation between Pb and Ca suggested a minor decrease in Pb concentration as a result of increasing Ca concentrations in TCLs of CTS patients. Unfortunately, no information on the Cd or Pb concentration relationship with Ca concentration in the TCL was available in the previous studies. Therefore, we discussed the relationship with ecotoxicological studies. Studies describing the relationship of Cd or Pb with Ca in human tissues and their correlation generally vary in the literature. The survey of Nowak and Chmielnicka [30] found that the increase in Pb concentration in hair causes a decrease in Ca concentrations. Others described the mechanism of Cd in the human bone to significantly reduce Ca absorption and excretion or/and exchange Ca in hydroxyapatite crystals, causing bone osteomalacia or osteoporosis [31]. Therefore, the same results can be observed in CTS patients. Also, the Ca content in the TCL did not appear to affect Cd or Pb deposit; this can be related to other factors.

## 5. Conclusions

As environmental toxicity studies with data-gap-filling strategies are used to assess risk and at the same time be encouraged to increase the performance of new human tests, this study presents a method that documents routine Ca, Cd, and Pb quantification in the TCL of humans with focus on high-resolution ICP-MS. The result was achieved and validated with linearity, LOD, LOQ, precision, and accuracy requirements. Although no studies measure calcification in TCL, ICP-MS enables testing of Ca concentration and potential for improving patient diagnosis. The results obtained above answered this study's question, and the contents of Ca in the TCLs of forty patients with CTS were not higher than the contents of Ca in the TCLs of control samples, indicating that the content of Ca in the TCL is not connected with CTS. The possible deposition of Pb and Cd with Ca content in the TCL was investigated, and the results showed no correlation between them as other factors could control this, and more studies are needed. The novel use of XRF for direct analyses of elements in the TCL was reported in this study. In this study, the determination was performed from a living human body, which enabled more diagnosis studies could be conducted.

## Figures and Tables

**Table 1:** Demographic characteristics of forty patients with CTS and three control volunteers with no CTS. After the questionnaire and full medical examination, all patients and volunteers were not diagnosed with other diseases.

Left-hand affected by CTS	Right-hand affected by CTS	Control with no CTS
1) age (years): 35.0 ± 0.3, number of patients: (*n* = 7), gender: female	2) age (years): 32.0 ± 0.2, number of patients: (*n* = 22), gender: female	3) age (years): 33 and 34, number of volunteers: (*n* = 2), gender: female
	4) age (years): 34.0 ± 0.6, number of patients: (*n* = 11), gender: male	5) age (years): 35 number of volunteers: (*n* = 1), gender: male

**Table 2:** Linear regression data of Ca, Cd, and Pb.

Parameter	Ca	Cd	Pb
Linear range	10–400 mM	10–400 *μ*M	10–400 *μ*M
*R* ^2^	0.99	0.99	0.99
Slope	207	994	3832
Intercept	45	22	2224
%Intercept	0.20	0.02	0.6

**Table 3:** RSD values for the precision of ICP-MS during the determination of Ca, Cd, and Pb in the lowest standard solution (10 mM, 10 *μ*M, and 10 *μ*M, respectively (*n* = 3 samples)).

Ca			
Day	Measured concentration (mM)	RSD value (%)	
		Repeatability	Intermediate precision

First	10.3 ± 0.04	0.4	
Fourth	9.35 ± 0.02	0.2	1.3
Sixth	10.6 ± 0.01	0.090	

Cd			
Day	Measured concentration (*μ*M)	RSD value (%)	
		Repeatability	Intermediate precision

First	9.40 ± 0.05	0.5	
Fourth	9.89 ± 0.08	0.8	1.0
Sixth	9.93 ± 0.03	0.3	

Pb			
Day	Measured concentration (*μ*M)	RSD value (%)	
		Repeatability	Intermediate precision

First	10.1 ± 0.01	0.1	
Fourth	9.94 ± 0.04	0.4	0.4
Sixth	10.2 ± 0.01	0.1	

**Table 4:** The spike recoveries of Ca, Cd, and Pb of the ddH_2_O. The determined concentrations were reported as the mean of three replicate experiments ± standard error of the mean (SEM).

Element	Concentrations (*μ*M)				
	Nominated concentration	Element concentration measured in a spiked ddH2O	Element concentration measured in an unspiked ddH_2_O	Recovery (%)	RSD (%)
Ca	50.0	47.3 ± 0.3	0.03 ± 0.0005	95 ± 0.02	0.63
Cd	50.0	49.6 ± 0.04	0.06 ± 0.0001	99 ± 0.01	0.080
Pb	50.0	46.7 ± 0.2	0.02 ± 0.0007	93 ± 0.03	0.40

## Data Availability

The data used to support the findings of this study are available from the corresponding author upon request.
